# Loneliness and Daily Stressors: The Protective Role of Daily Positive Events

**DOI:** 10.1093/geroni/igaa057.2186

**Published:** 2020-12-16

**Authors:** Joanna Hong, Susan Charles, Nancy Sin, David Almeida

**Affiliations:** 1 University of California, Irvine, Irvine, California, United States; 2 BSS, Irvine, California, United States; 3 University of British Columbia, Vancouver, British Columbia, Canada; 4 Pennsylvania State University, University Park, Pennsylvania, United States

## Abstract

Lonely individuals are particularly vulnerable to daily stressors. Yet, less is known about the protective role of daily positive social events on days lonely individuals experience a stressor. The current study examined whether experiencing a positive social event on the same day as a stressor helps lonely individuals maintain their daily emotional well-being. Participants from the Midlife in the United States Survey II and the National Study of Daily Experiences II reported their trait-levels of loneliness. A subset of the participants (N=1,730) also completed eight days of daily interviews and reported experiences of stressful events, positive social events, and emotions. On days lonely individuals reported experiencing a stressor, experiencing a positive social event was associated with a less increase in daily negative affect. However, this buffering effect did not generalize to non-lonely individuals. Results highlight a protective asset that might be important for helping lonely individuals maintain daily well-being.

